# Epigenome-wide association studies: current knowledge, strategies and recommendations

**DOI:** 10.1186/s13148-021-01200-8

**Published:** 2021-12-04

**Authors:** Maria Pia Campagna, Alexandre Xavier, Jeannette Lechner-Scott, Vicky Maltby, Rodney J. Scott, Helmut Butzkueven, Vilija G. Jokubaitis, Rodney A. Lea

**Affiliations:** 1 Department of Neuroscience, Central Clinical School, Monash University, Melbourne, Australia; 2 Centre for Information Based Medicine, Hunter Medical Research Institute, Newcastle, Australia; 3 School of Biomedical Sciences and Pharmacy, University of Newcastle, Newcastle, Australia; 4 Department of Neurology, Division of Medicine, John Hunter Hospital, Newcastle, Australia; 5 Division of Molecular Medicine, New South Wales Health Pathology North, Newcastle, Australia; 6 Department of Neurology, Alfred Health, Melbourne, Australia; 7 Centre for Genomics and Personalised Health, School of Biomedical Sciences, Queensland University of Technology, Brisbane, Australia

**Keywords:** Epigenetics, Methylation, EWAS, ChAMP, Complex diseases, Bioinformatics

## Abstract

The aetiology and pathophysiology of complex diseases are driven by the interaction between genetic and environmental factors. The variability in risk and outcomes in these diseases are incompletely explained by genetics or environmental risk factors individually. Therefore, researchers are now exploring the epigenome, a biological interface at which genetics and the environment can interact. There is a growing body of evidence supporting the role of epigenetic mechanisms in complex disease pathophysiology. Epigenome-wide association studies (EWASes) investigate the association between a phenotype and epigenetic variants, most commonly DNA methylation. The decreasing cost of measuring epigenome-wide methylation and the increasing accessibility of bioinformatic pipelines have contributed to the rise in EWASes published in recent years. Here, we review the current literature on these EWASes and provide further recommendations and strategies for successfully conducting them. We have constrained our review to studies using methylation data as this is the most studied epigenetic mechanism; microarray-based data as whole-genome bisulphite sequencing remains prohibitively expensive for most laboratories; and blood-based studies due to the non-invasiveness of peripheral blood collection and availability of archived DNA, as well as the accessibility of publicly available blood-cell-based methylation data. Further, we address multiple novel areas of EWAS analysis that have not been covered in previous reviews: (1) longitudinal study designs, (2) the chip analysis methylation pipeline (ChAMP), (3) differentially methylated region (DMR) identification paradigms, (4) methylation quantitative trait loci (methQTL) analysis, (5) methylation age analysis and (6) identifying cell-specific differential methylation from mixed cell data using statistical deconvolution.

## Introduction

Epigenetic mechanisms involve modifications to genomic DNA (both heritable and/or modifiable) that can affect cellular phenotypes and in turn, influence complex disease aetiology and outcomes. The most widely studied epigenetic mechanism is DNA methylation, which can regulate gene expression through the presence or absence of a methyl group on cytosine-phosphate-guanine (CpG) dinucleotides. Over the last decade, the ability to study methylation at the genome-wide level has led to the application of the epigenome-wide association study (EWASes), which has increased our understanding of the role of methylation in many diseases [1–4]. As genome-wide methylation scanning technology has evolved, so too have bioinformatic tools to process, analyse and interpret methylation data from EWASes.

The aim of this review is to perform an up-to-date critical assessment of the tools and strategies available for conducting EWASes, specifically focusing on blood-cell derived methylation data. We focus on blood cells because (1) peripheral blood is one of the least-invasive tissues to collect, (2) there are many large-scale DNA banks of convenience available for conducting EWASes, (3) the most common publicly available methylation data is from whole blood or whole blood cell subsets, and (4) blood cell pathology is involved in many complex diseases. Furthermore, we review study design and methodological features of EWASes that have not been addressed in the literature previously: (1) longitudinal study designs, (2) the chip analysis methylation pipeline (ChAMP), (3) differentially methylated region (DMR) identification paradigms, (4) methylation quantitative trait loci (methQTL) analysis, (5) methylation age analysis and (6) identifying cell-specific differential methylation from mixed cell data using statistical deconvolution. The goal of this review is to provide researchers with a reference workflow and guidelines for conducting blood-cell-based EWASes.

## Epigenome-wide association studies

The aim of an epigenome-wide association study (EWAS) is to examine genome-wide epigenetic variants (predominantly DNA methylation at CpGs), to detect differences that are statistical associated with phenotypes of interest.

The most common way to study DNA methylation is with bisulphite converted genomic DNA and microarrays. Bisulphite conversion deaminates unmethylated cytosines, producing uracil, on denatured genomic DNA. Methylated cytosines remain unaffected, and therefore, bisulphite converted genomic DNA contains methylated cytosines only. Microarrays are a collection of different oligonucleotides fixed on a solid substrate (usually glass) that can hybridise to complementary DNA strands. The methylation level is measured at each CpG present on the microarray and compared between (or within) groups of interest to detect differentially methylated positions (DMPs) and regions (DMRs). A DMP is a single CpG dinucleotide that is differentially methylated between groups, as determined by statistical significance and effect size thresholds. The definition of a DMR differs between studies based on the algorithm used but can broadly be defined as a region containing multiple DMPs.

**Fig. 1:**
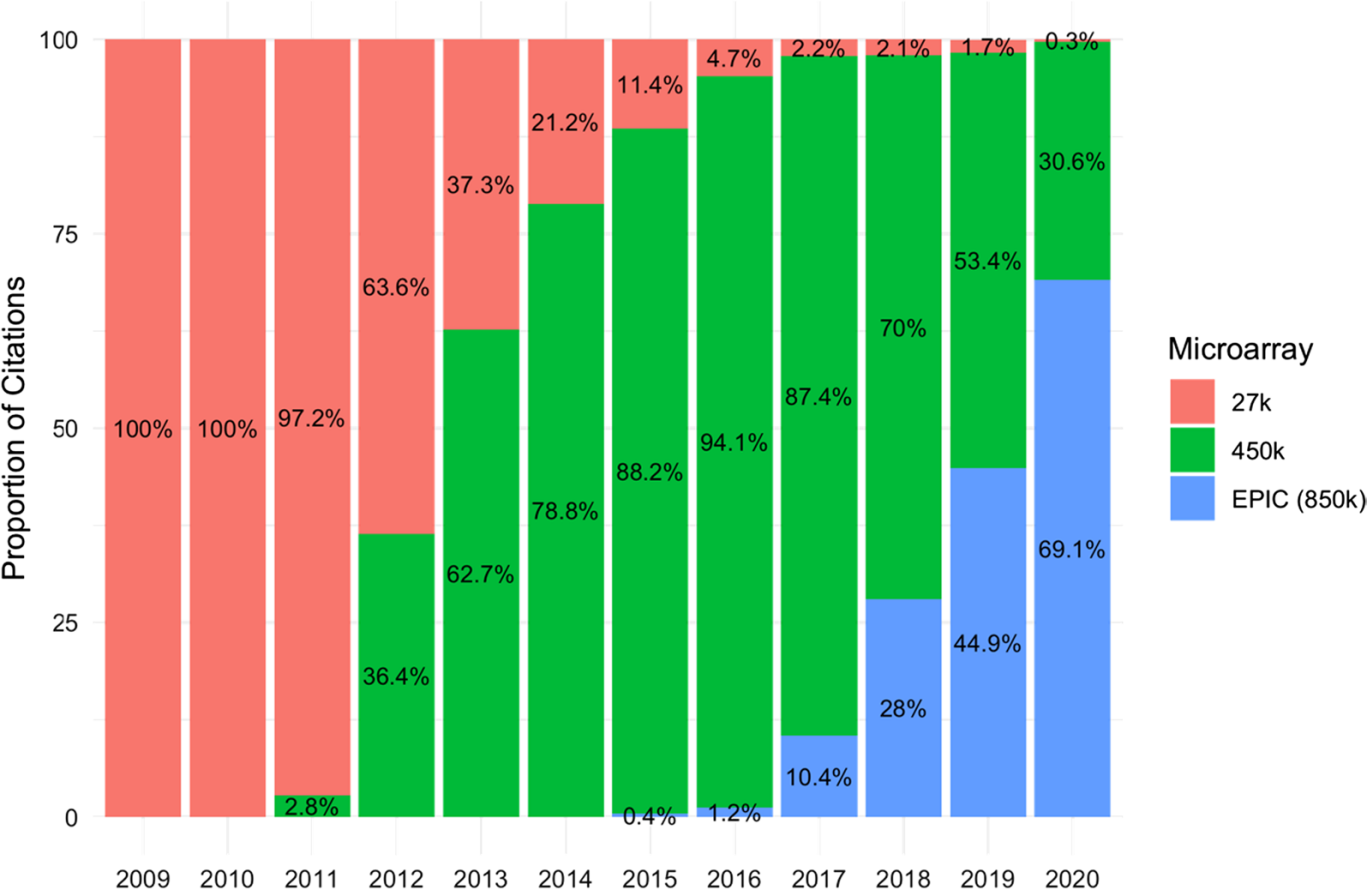
Popularity of methylation microarrays. The proportion of EWASes deposited on GEO (NCBI) each year, by array type. Abbreviations*:* EWAS = Epigenome-wide association study, GEO = Gene Expression Omnibus, NCBI = National Center for Biotechnology Information

The first commercial high-density microarray measuring genome-wide methylation was the HumanMethylation27 (27K) released by Illumina in 2009 [5]. The 27K microarray allowed researchers to measure methylation across more than 27,000 CpG sites spanning over 14,000 genes and paved the way for mainstream EWASes. The HumanMethylation450 (450K) microarray followed in 2011 and rapidly gained popularity as it measures methylation at over 450,000 CpG sites [6]. The 450K remains the most cited Illumina microarray for DNA methylation studies to date (Fig. 1). In 2016, Illumina produced a new iteration of the 450K, called the HumanMethylation850 (EPIC) microarray, which measures methylation at over 850,000 CpG sites [7]. Illumina microarrays predominantly measure methylation in gene promoter regions. The coverage of intergenic regulatory regions has improved with each iteration of the microarray; however, the EPIC microarray still only covers 58% of FANTOM enhancers, 27% of proximal regulatory elements and 7% of distal regulatory elements [7]. Notably, Agilent now produces microarrays that measure methylation at over 235,000 CpG sites; however, these microarrays are not cited as frequently as Illumina microarrays. Nimblegen also produced microarrays, but these were discontinued in 2012.

**Fig. 2:**
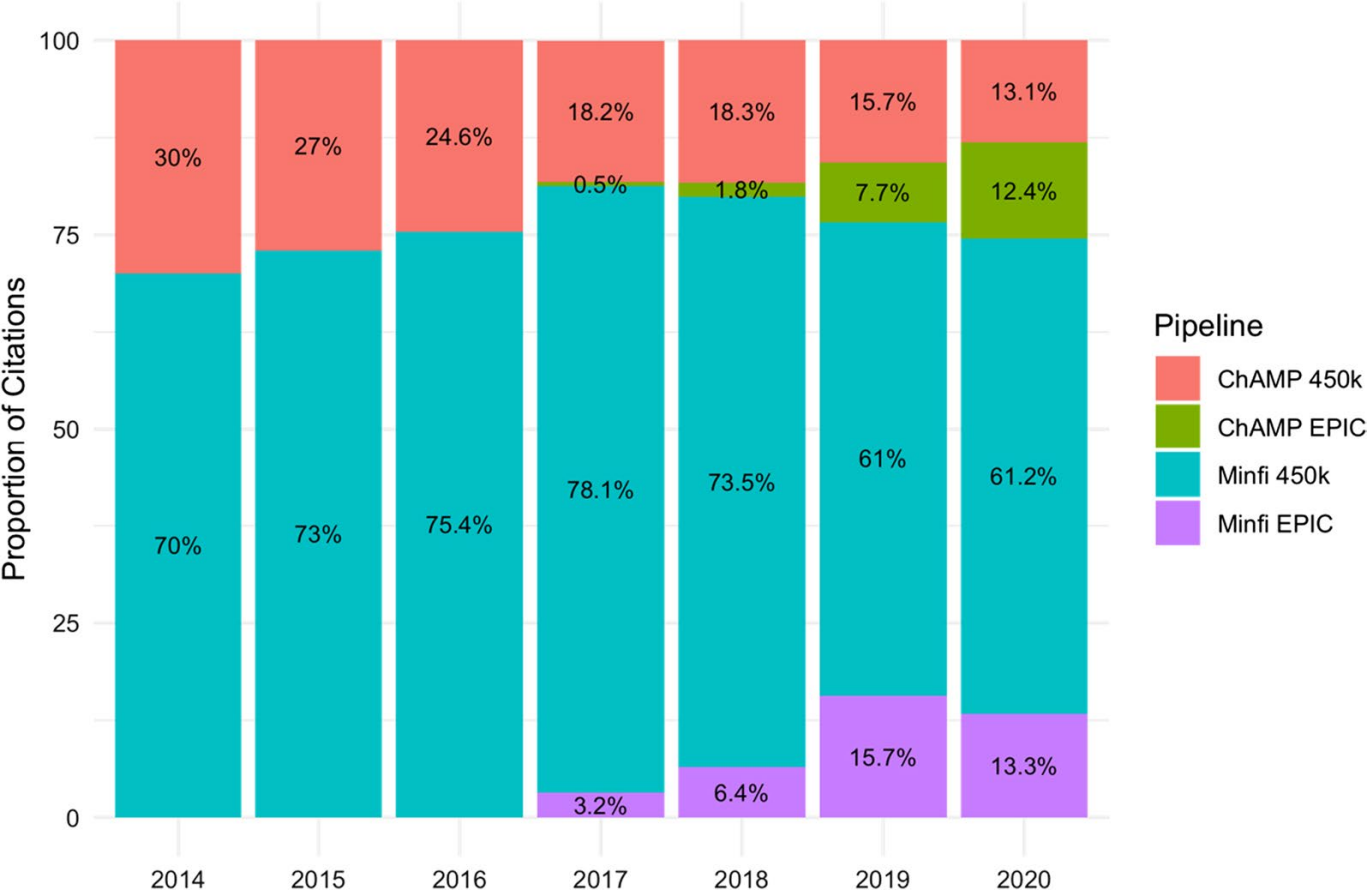
Popularity of EWAS pipelines. The proportion of PubMed citations by year for methylation data analysis using different Illumina arrays. Abbreviations: EWAS = Epigenome-wide association study

**Fig. 3:**
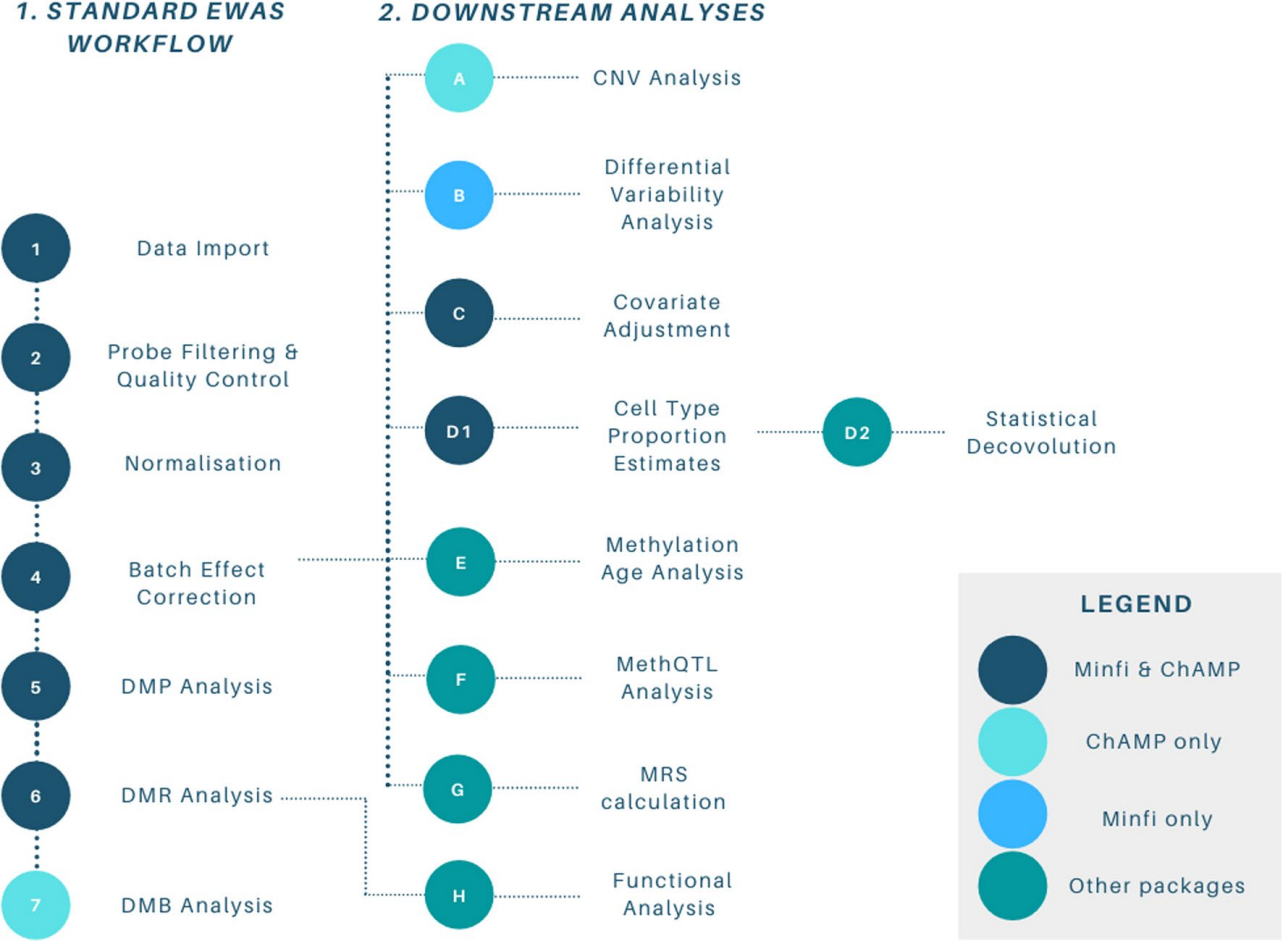
A standard EWAS workflow using *Minfi* or *ChAMP* packages. Analyses are either common to both packages, specific to one package, or completed with other unspecified packages. Abbreviations: DMP = differentially methylated position, DMR = differentially methylated region; DMB = differentially methylated block; CNV = copy number variation; methQTL = methylation quantitative trait loci; MRS = methylation risk score

The ability to measure methylation in a high-throughput manner drove the development of bioinformatic pipelines. These have streamlined analyses and overcome the hurdles that arise from highly dimensional datasets. Two main bioinformatics analysis packages for EWAS data are *Minfi* [8] and *ChAMP* [9], which emerged as open-source alternatives to *GenomeStudio*, the original proprietary tool provided by Illumina. Both packages were released in 2014 and were built to analyse 450k microarray data. In 2017, they were updated to also include EPIC microarray data [10, 11]. *Minfi* is the most cited tool for 450k data analysis, while *ChAMP* is becoming the most cited tool for EPIC data analysis (Fig. 2). *Minfi* and *ChAMP* allow users to import data files directly produced from methylation microarrays (i.e. raw.idat files), perform quality control (QC), normalisation and detection of both DMPs and DMRs. Different downstream analyses are available for each package (Fig. 3).

## Study designs

EWASes can be conducted using unrelated case–control and longitudinal designs, as well as one sample quantitative trait and family-based study designs. Here, we compare and contrast case–control and longitudinal study designs and discuss other important considerations such as power and sample size. Quantitative trait and family-based study designs differ substantially from those of non-related individuals and have been reviewed in detail previously [12]. The main difference between case–control and longitudinal study designs is the practicality and affordability of case–control studies in comparison with longitudinal studies. However, only longitudinal studies can assess the relationship between changes in epigenetics and phenotype during the course of disease. Notably, case–control studies investigate the relationship between dichotomous traits and methylation using contrast comparisons.

### Case–control designs

Case–control EWASes are the most employed study design. The case–control design is a standard design in epidemiology and involves grouping unrelated participants into a phenotype of interest, such as the presence of disease, and compares CpG methylation levels to a group of subjects without the phenotype (i.e. a control group) [13–16]. The control group can be matched for potential confounding factors such as age, sex, ethnicity or genotype at a locus that has previously been associated with the phenotype of interest. Subjects are usually collected in a cross-sectional manner from the population of interest making this design the most feasible in terms of logistics and cost (discussed below in "Longitudinal designs" section). For blood-based EWAS, researchers can utilise existing DNA biobanks from past case–control genome-wide association studies (GWASes) [17]. Thus, the main benefit of this study design is the ability to obtain large subject numbers.

The primary limitation of the case–control study design is the inability to determine the timing of the relationship between differential methylation and phenotype. That is, whether differential methylation between cases and controls results in, or is a result of, disease. For this reason, case–control EWASs are typically restricted to claims to association rather assigning cause or effect of the relationships. Auxiliary approaches such Mendelian randomisation (MR) can be used within the case–control designs to statistically infer cause and effect between CpGs associated with the phenotype. MR uses genetic variants that are associated with the same CpGs and/or the phenotype to reveal potential causal associations [18]. However, prospective longitudinal study designs are required to truly understand the timing and mechanism behind phenotype-inducing changes in methylation.

### Longitudinal designs

Longitudinal studies allow researchers to determine intra-individual trajectories (changes) as well as inter-individual variability in methylation and/or phenotype over time. However, they are more difficult to set up than case–control studies. These studies follow groups of individuals over months, years or decades measuring methylation, and if possible, phenotype, at multiple timepoints. The commonest form of longitudinal studies in the EWAS literature are natural history studies, which track methylation trajectories from birth in healthy individuals [19–24]. However, it is harder to establish longitudinal studies following disease states, as pre-disease onset samples are very difficult to collect. A significant advantage of the longitudinal study design is the ability to track methylome changes in relation to time and phenotype, possibly allowing causal relationships to be established. Nevertheless, the time and cost associated with longitudinal studies remain prohibitive for many researchers. Consequently, the majority of longitudinal EWASes study healthy, natural history cohorts.

Longitudinal studies in natural history cohorts demonstrate the dynamic nature of DNA methylation throughout the lifespan, particularly in the early years of life. During the first five years of life, the methylome undergoes drastic remodelling with a tendency towards global hypermethylation [19–24]. Methylation changes predominantly occur on autosomal chromosomes [19–23], with hypermethylation in CpG dense regions, including gene promoters, intragenic regions and transcription start sites [20–23]. Hypermethylated genes are overrepresented in developmental functions such as tissue morphogenesis, haematological system development, the effector immune response, neuronal-related functions and cell–cell signalling [21–23]. Hypomethylation occurs in CpG sparse regions [20, 22, 23], primarily in immune response-related genes including antigen binding and intracellular signalling, cellular components related to the major histocompatibility complex (MHC) protein complex and cytoskeleton, as well as messenger RNA and protein metabolism [21–23]. Notably, leukocytes undergo major epigenome remodelling in the first five years of life, potentially indicating an “immunological window of opportunity” in childhood [22, 25]. In individuals over 50 years of age, inter-individual variation and intra-individual demethylation tend to increase with time [19, 26–28]. Age-related demethylation is particularly pronounced at Alu repetitive elements (conserved regulatory regions), which correlate with genome-wide methylation levels [27].

Longitudinal study designs have also been used to study epigenetic drift in monozygotic (MZ) and dizygotic (DZ) twin pairs. In twins, epigenetic drift refers to the diverging epigenomes over time, as they are exposed to different environmental factors [15–18], and is more pronounced in DZ than MZ twins [28–31]. As MZ twins are genetically identical, they are useful for studying the impact of environmental factors on the epigenome over time. Such studies have shown that genetics explains less than 24% of variation in epigenetic drift, demonstrating a substantial impact of environmental factors on the epigenome [29]. Epigenetic drift also attenuates with age, and primarily affects genes enriched for immune and inflammation pathways [29]. In summary, genetics contributes to the methylome's stability over time, while environmental factors contribute to epigenetic drift.

In the current EWAS literature, longitudinal studies typically examine changes in methylation across two time points only. As such, methodological strategies for conducting studies with multiple time points are lacking. Furthermore, these studies have used data derived from the 450k microarray, which is enriched for CpG-dense genomic regions (CpG islands). There is growing evidence that methylation in CpG-sparse regions, such as enhancers and gene bodies, has significant functional consequences through altering gene expression [32]. Hence, longitudinal studies with multiple time points using the EPIC microarray could provide novel insights into methylation trajectories in health and disease.

### Replication or validation analyses

Replication or validation analyses are critical for EWASes to confirm preliminary findings. This is particularly pertinent in epigenetic studies as a range of confounding environmental exposures—known and unknown—may be present. As with GWASes, independently ascertained replication cohorts are required to confirm (or refute) preliminary results and establish effect sizes, and therefore inform pathology or clinical utility (e.g. biomarkers). Replication is defined as the reproducibility of preliminary results in a cohort that is as similar to, but independent of, the preliminary cohort. For this reason, it is often difficult—and sometimes impossible—for researchers to obtain a suitable replication cohort. This is especially true for rare diseases where patient numbers are difficult to build up. In this instance, there are several approaches researchers can take to *validate* preliminary results. Validation is defined as corroboration of results in a cohort, or using a dataset, that does not originate from the discovery phase of the study. In EWASes, validation can be achieved by (1) corroborating preliminary findings in a similar, although not identical cohort, or general population, (2) confirming that preliminary findings are not corroborated in a healthy or natural history cohort or dataset, indicating disease specificity, (3) utilising EWAS databases (see "Epigenome-wide association study databases" section) to access raw.idat files and/or summary statistics for validation analyses, (4) using the literature to provide biological or pathological support for preliminary results or (5) using animal studies to gain specific mechanistic insight. For animal studies, rodents are useful as they age faster than humans and researchers can control for environmental exposures and confounders.

### Power and sample size considerations

EWASes of complex diseases must be statistically powered to identify modest, but important, differences in DNA methylation between groups or time points (i.e. effect sizes). Statistical power is defined as the probability that a statistical test rejects the null hypothesis when the alternative hypothesis is correct. Therefore, in adequately powered studies, there is a reduced risk of type I (false positive) and type II (false negative) errors. Significance thresholds, sample size and effect size all affect statistical power of EWASes.

In EWASes, Type I error rates (significance thresholds) are impacted by multiple testing as a vast number of CpG sites are analysed. Bonferroni corrected significance thresholds are sometimes used to overcome the multiple testing burden. However, Bonferroni deflation for the number of CpG sites tested is widely considered to be too stringent in EWASes as methylation is highly correlated across the genome. Thus, the actual number of independent tests is far fewer than the number of CpGs on each microarray [33]. Therefore, a false-discovery rate (FDR) threshold is commonly used, which provides a balanced compromise between type I and type II error rates (i.e. FDR < 0.05). However, the heteroskedasticity of methylation data (i.e. non-constant variance of methylation levels between groups) and non-uniform distribution of p-values across measured CpG sites can violate FDR assumptions. Thus, using an FDR threshold could limit the reproducibility of results across studies [33]. To overcome the limitations of both Bonferroni corrected p-values and FDR thresholds, simulation studies have calculated the number of independent tests in EWASes using methylation data from the 450k and EPIC microarrays. An unadjusted significance threshold of 9.42 × 10^−8^ (95% CI = 2.97 × 10^−8^–1.49 × 10^−15^) is recommended for analyses using EPIC data [33], and 2.4 × 10^−7^ (95% CI not reported) for analyses using 450k data [34, 35]. The ChAMP pipeline (outlined below in "The chip analysis methylation pipeline (ChAMP)" section) calculates unadjusted and adjusted p-values (FDR), allowing researchers to use the significance threshold that is most suitable for their study.

As complex phenotypes rarely have effect sizes larger than 5%, the most straightforward way to increase power is to increase sample size. EWAS power studies using case-control [33–35] or family-based [34, 35] simulations recommend a sample size of 1000 (500 cases and 500 controls) to detect statistically significant DMPs and DMRs. However, in some cases these are not required. For example, due to large methylation differences in the Human Leukocyte Antigen (HLA) region, several EWASes of multiple sclerosis have identified differential methylation of > 20% between cases and controls with sample sizes of 20–30 per group [13–15]. There are currently no published sample size recommendations for longitudinal EWASes. However, longitudinal studies in Parkinson’s disease and Type I diabetes have identified differential methylation between groups with sample sizes ranging from 85 to 190 per group [36, 37]. Large sample sizes may be difficult to obtain in longitudinal studies, due to costs and attrition rates.

## The chip analysis methylation pipeline (ChAMP)

**Fig. 4:**
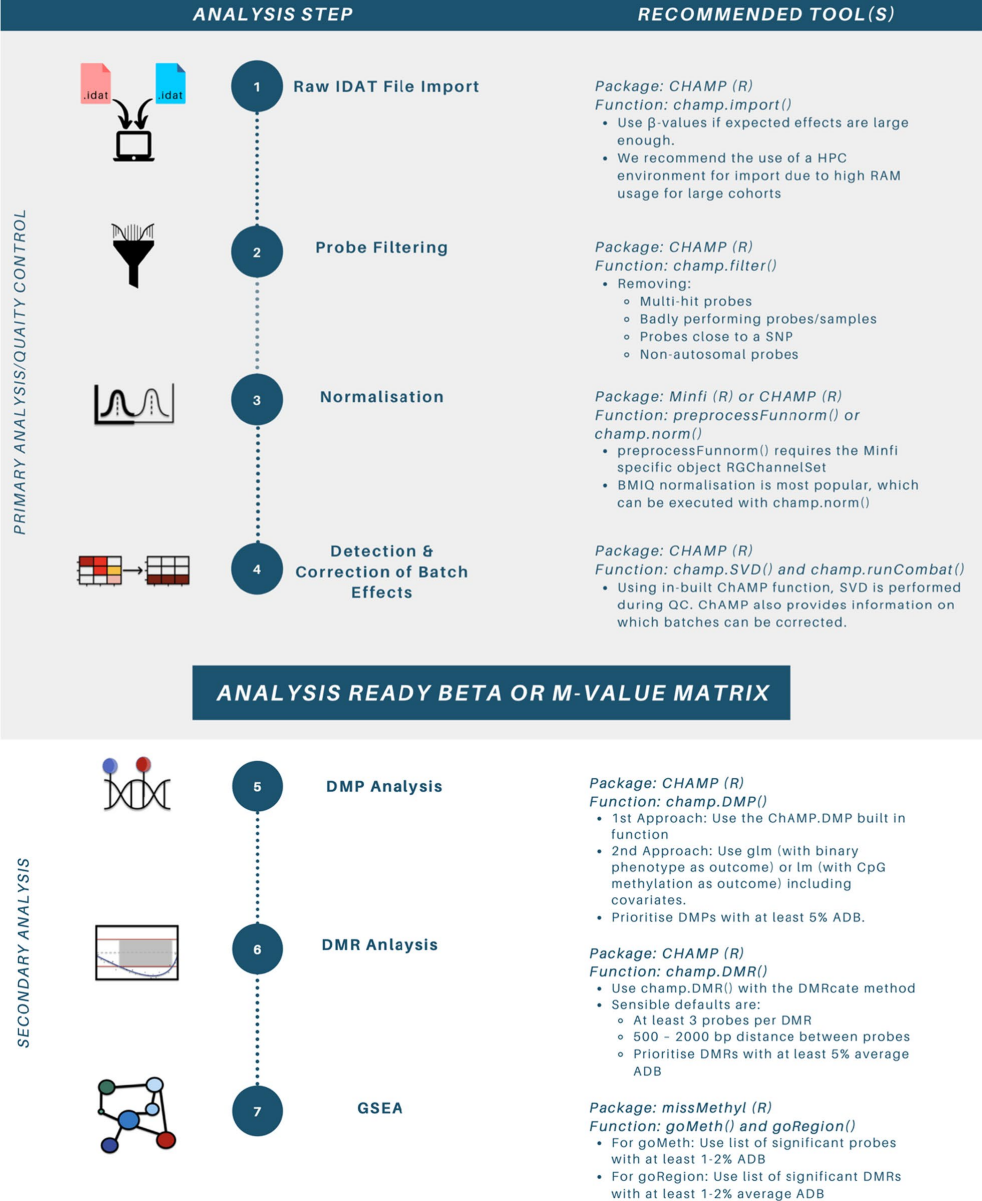
Steps and tools for primary EWAS analysis steps. Listed tools include ChAMP (https://bioconductor.org/packages/release/bioc/html/ChAMP.html), Minfi (https://bioconductor.org/packages/release/bioc/html/minfi.html) and missMethyl (http://bioconductor.org/packages/release/bioc/html/missMethyl.html). Abbreviations: EWAS = epigenome-wide association study, ChAMP = chip analysis methylation pipeline, HPC = high performance computer, RAM = random access memory, SNP = single nucleotide polymorphism, BMIQ = beta mixture quantile, SVD = singular value decomposition, QC = quality control, DMR = differentially methylated region, ADB = absolute deta beta

*ChAMP* is a Bioconductor package that provides a powerful tool for analysis of DNA methylation data obtained with the Illumina 450K or EPIC microarrays [11]. It is designed for the R statistical environment [11]*,* and integrates existing pre-processing and analysis tools, such as *Minfi* [8], into a single pipeline. The *ChAMP* pipeline consists of eight main functions, which can be executed in full with the command *champ.process*(). Nevertheless, we recommend completing each function separately so that researchers can assess the interim results and tailor the parameters of each function to best suit their analysis [11]. The steps and recommended tools for primary EWAS analyses discussed below are summarised in Fig. 4.

*ChAMP* is designed for case–control designs, i.e. identifies differential methylation between two categorical groups. This is also adequate for case–control studies and longitudinal studies with two time points. However, *ChAMP* is not a suitable package for studies of multiple groups including longitudinal studies with more than two time points. For such analyses, researchers will need to use the individual packages incorporated into *ChAMP*, such as *limma* for DMP analysis and *DMRcate* for DMR analysis. *ChAMP* is also unsuitable for studies of continuous phenotypes. There are currently no purpose-built tools for this type of analysis; therefore, researchers will need to use alternate statistical approaches, such as linear regression modelling between CpG and phenotype within a suitable statistics program (e.g. R environment [38]).

### Data pre-processing

#### Probe filtering

*ChAMP* offers two functions to load data from.idat files and a corresponding sample sheet: *champ.import*() and *champ.load*(). The former uses *Minfi* functions to return objects (e.g. data matrices) fed directly into downstream normalisation functions [8], while the latter imports and filters data in one step [11]. Alternatively, researchers can use *champ.filter()* to customise the following parameters: (1) detection p-values (*p* > 0.01), (2) low-quality probes (< 3 beads in ≥ 5% of samples per probe), (3) probe type (non-CpG probes), (4) chromosomal location (non-autosomal), (5) presence of single-nucleotide polymorphisms (SNPs) in the probe sequence (population-specific) [5], (6) cross-hybridisation [39] and (7) multi-hit probes (probes with unspecific genomic alignment) [40]. Default filtering thresholds are adequate for most analyses. The dataset used for cross-hybridised probe filtering with *ChAMP* is from an early study which interrogated the 450k microarray [40]. More recently, ~15,000 additional cross-hybridised probes were identified for the EPIC microarray [41]. Therefore, it is imperative that researchers using the EPIC microarray further filter data for the additional cross-hybridised probes prior to data normalisation.

#### Beta- and M values

*ChAMP* accommodates the use of either beta- or M values representing CpG methylation. Beta-values are an index of methylation levels and range from zero (completely unmethylated) to one (completely methylated). Notably, methylation beta-values differ to beta coefficients produced by regression models. Beta-values are the ratio of the methylated probe intensity and overall probe intensity (the sum of methylated and unmethylated probe intensities). Methylation beta-values are easy to interpret as they can be converted to a percentage of methylation ranging from 0 (unmethylated) to 100% (methylated). One drawback of beta-values is that they follow mixed statistical probability distributions (i.e. beta-binomial), which can cause issues for conventional linear regression models whereby CpG beta-values are modelled as the dependent variable. For this reason, beta-values may be transformed into M values, which are the log^2^ ratio of the methylated probe intensity and the unmethylated probe intensity. Negative M values indicate lower methylation, whereas positive M values indicate higher methylation. Although M values are considered statistically more appropriate than beta-values when the CpG is modelled as the dependent variable than beta-values, they lack intuitive interpretation [42]. Detailed assessment of the use of beta- or M values has been published elsewhere [42].

#### Probe imputation

Imputation is the process of inferring beta-values for missing probes in a beta matrix using statistics and machine learning. Missing probes can result from inadequate assays or across microarray iterations. For example, probes from the 450K microarray compared to the EPIC microarray can be imputed for direct comparison. Imputation is particularly useful in longitudinal studies to harmonise data collected with different platforms across timepoints due to technological development.

*champ.impute()* imputes missing beta-values in a filtered beta matrix (i.e. after *champ.load())* via three methods: removal of the missing probes, k-nearest neighbours or a combination of both [11]. K-nearest neighbour is a machine learning method in which a Euclidean metric is used to identify neighbouring probes (to the missing probe), and the beta-value of the missing probe is imputed as the average of the beta-values at the neighbouring probes [11]. We recommend the removal of missing probes for small sample sizes and a combination of removal and k-nearest neighbours for larger sample sizes.

#### Data normalisation

**Fig. 5:**
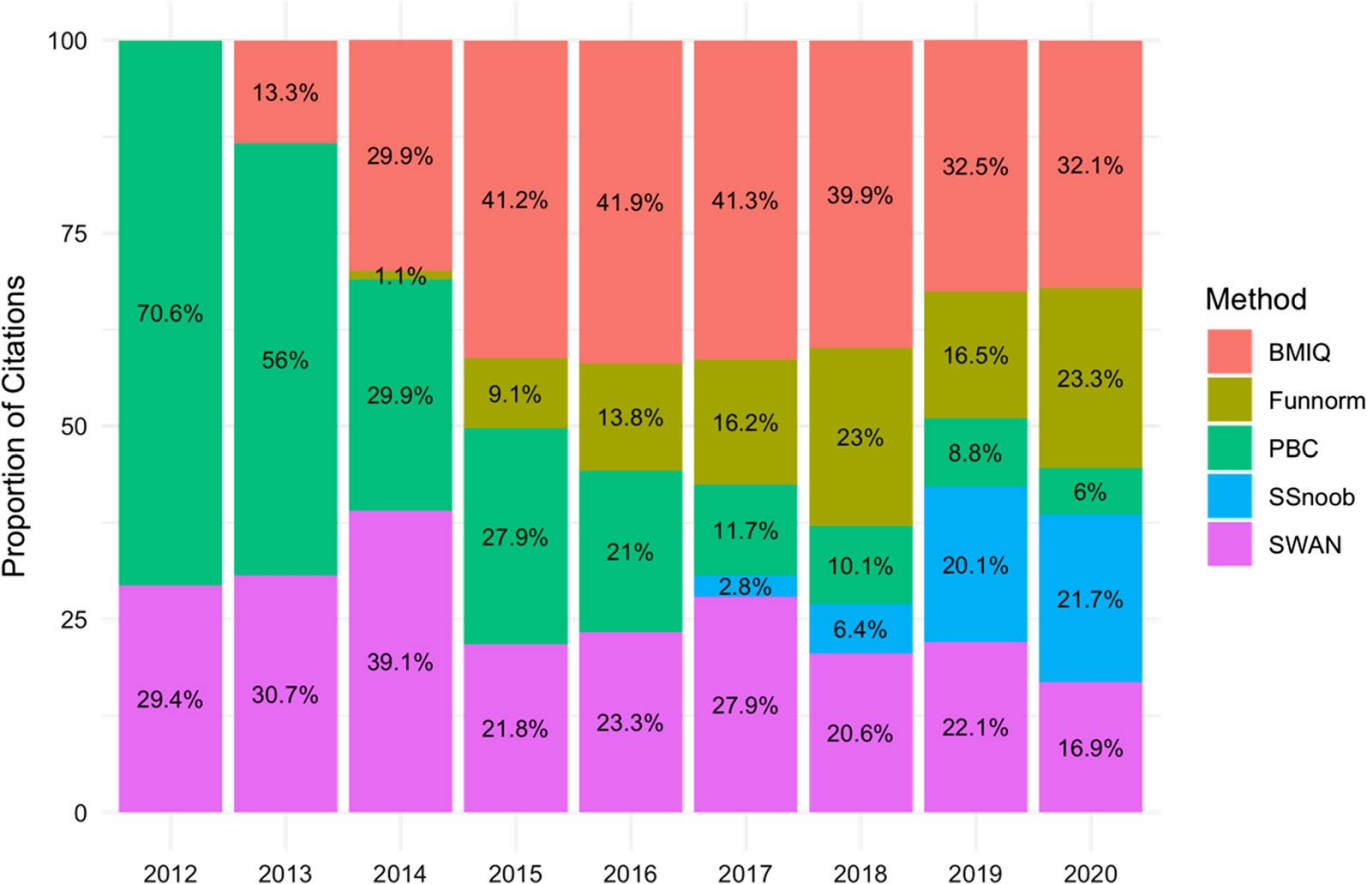
Popularity of normalisation methods. The proportion of PubMed citations by year for methylation data normalisation algorithms.

Illumina microarrays use two distinct hybridisation chemistries to measure methylation at probes, Type I and Type II, which produce different beta-value distributions. These distributions must be normalised to avoid the biased detection of DMPs enriched for Type I probes. Beta-mixture quantile normalisation (BMIQ) [43] is the default normalisation method in *ChAMP*, but other options are available, including subset-quantile within microarray normalisation (SWAN) [44], peak-based correction (PBC) [45] or functional normalisation (Funnorm) [46]. All of these methods have been previously described in detail [47–51]. For analyses where there is a strong association between methylation and phenotype, each method performs similarly in terms of accuracy [50, 51]. However, Funnorm produces the most replicable results in analyses where global methylation changes are expected; for example, in case–control, between-disease studies or inter-tissue comparisons. The same method also removes a large amount of technical variation in the unsupervised normalisation process, improving downstream batch effect correction. Nevertheless, BMIQ remains the most popular normalisation method to date (Fig. 5).

It is not uncommon for researchers to use methylation data derived from different microarrays. For example, in case–control studies, cases may be prospectively recruited and analysed using the EPIC microarray, while historical 450k microarray data may be used for controls. In longitudinal analyses, the 450k microarray may be used at baseline, while the EPIC microarray is used at follow-up timepoints. In this scenario, the single-sample noob (*SSnoob*) function in *Minfi* is the most useful tool for normalisation [10]. *SSnoob* integrates 450K and EPIC microarray data for joint normalisation, outperforming BMIQ and Funnorm in this process [10].

#### Addressing technical variation (batch effects)

The default method for batch effect identification in *ChAMP* is singular value decomposition (SVD) [9]. Basically, SVD correlates principal components with biological and technical variation using information from the user-provided sample sheet [25].

The default method for batch effect correction in *ChAMP* is *ComBat* [9], which uses an empirical Bayes method to correct batch effects. *ComBat* requires two inputs: (1) the variables which should not be adjusted (i.e., biological variation caused by the variable of interest), and (2) the batch variables to be adjusted for (for example, slide, microarray, plate), previously identified in SVD analysis. Researchers should be aware that *ComBat* can introduce false signals if biological and technical variation are partially or wholly confounded [52–55]. In a case–control study, this may occur if all samples from cases run on the same plate and controls on another, thus causing systematic technical error via batch effects. The analogous error in a longitudinal study is running samples from each time point on different plates. To reduce the risk of false signals, *ChAMP* checks that technical and biological variation is not confounded before adjustment [11]. False signals can be minimised a priori by manually randomising samples across microarrays and excluding biological covariates from batch effect correction [55]. In an ideal experiment, all samples would be processed in one batch; however, this is impossible for large sample sizes. The nature of longitudinal study designs means that they are at higher risk of batch effects than case–control studies, as samples are collected and processed at different time points. Thus assays, microarrays and techniques may evolve, leading to technical variation. *ComBat* has been shown to be effective in correcting technical variation in longitudinal gene expression studies [56]. Biological and clinical covariates likely influence methylation patterns and should be addressed in downstream statistical analyses rather than at the data pre-processing stage [55]. Notably, for researchers correlating methylation data with other clinical datasets, *ComBat* has utility in correcting batch effects in various datasets, including gene expression and imaging data [57–59]. Alternatively to *ComBat*, the *missMethyl* package uses Remove Unwanted Variation (RUV). The RUV uses negative controls on the microarray to identify and remove unwanted variation [60, 61]. As a rule of thumb, it is reasonable to move onto downstream analyses if the first two principal components identified with SVD explain more than 80% of the covariance in the data, and if these are adequately corrected for prior to progressing [11].

### Statistical association testing

The statistical association between methylation levels (beta- or M values) and phenotype are identified at the level of individual CpG sites (DMPs) and broader genomic regions (DMRs). Typically, DMP analyses are straightforward association tests between methylation beta-values and phenotype, whereby the specific statistical test used will depend on the study design and cohort characteristics. DMR analysis is more complex and as such, multiple algorithms for DMR detection are available.

#### Differentially methylated positions (DMPs)

DMPs are CpG sites with statistically significant differences in average (usually mean) methylation levels between groups. *ChAMP* can identify DMPs between two categorical groups of interest: case–control studies, and longitudinal studies with two time points. For longitudinal studies that collect data for more than two time points, there are different ways of approaching analyses, to identify intragroup changes between time points and/or intergroup differences over time. For the former, methylation data from the same group at two time points can be analysed as if it was comparing two separate groups using general linear models with repeated measures. For longitudinal studies with more than two time points, linear mixed models (LMMs) including time as a random effect term can be used to compare methylation at each timepoint simultaneously. LMMs are useful in longitudinal studies where missing data is common, or to address differences in the timing of measurements between groups. To identify intergroup differences in change over time there are multiple statistical approaches available: (1) use *ChAMP* to identify DMPs at baseline and re-analyse methylation differences at only these sites at subsequent time points to identify the methylation trajectory (change) at these sites over time, (2) use *ChAMP* to identify DMPs at each timepoint independently, and correlate results between timepoints, noting which DMPs are variable (significantly different at any time point) or consistent (significantly different at each time point), and their direction of effect or (3) use LMM with a group contrast term to assess the relationship between change in methylation over time and phenotype group.

The *champ.DMP()* function in *ChAMP* uses the Bioconductor package *limma* [62] to identify DMPs from a beta-matrix. This package conducts a pairwise comparison of beta-values between groups, by fitting the same general linear model to each probe separately and computing a moderated t-statistic and unadjusted *p* value. A moderated t-statistic is the ratio of the beta-value for a CpG to a pooled standard error (SE). By pooling information from all CpGs *limma* moderates SE at individual probes to improve inference about each CpG. *ChAMP* subsequently corrects unadjusted *p* values for multiple testing using the Benjamini and Hochberg method [63] and reports an FDR. The use of unadjusted and adjusted significance values by *ChAMP* allows researchers the option of significance thresholds to guide the interpretation. However, strict adherence to significance thresholds may lead researchers to overlook DMPs with significant biological effects (i.e. incur Type II error). Therefore, if few DMPs surpass default significance thresholds, we recommend the use of secondary criteria applied in a stepwise manner: (1) identify DMPs with an unadjusted *p* value below 0.05, (2) identify DMPs with effect sizes larger than 10%, (i.e. an absolute delta beta-value > 0.1). If there are still no, or few, hits researchers may then relax the effect size threshold further in the interests of detecting minor effect DMPs. Studies show < 1% variation in beta-values across technical replicates using the EPIC microarray [6]. This suggests that at least some CpGs with effect sizes below this will be affected by technical variation, rather than biological variation. As such, we recommend a conservative approach of removing DMPs with effect sizes below 2%. However, reducing the threshold to 1% may still be adopted if desired and yield some important, albeit modest, biological insights. Numerous published studies have used these secondary criteria to identify methylation differences between cases and controls in various diseases and cell types [13–15, 64].

Covariates (e.g. age and sex) can be included in DMP analysis as secondary analyses run outside of *ChAMP*, using packages like *limma or* base R functions. We recommend against including covariates in the primary DMP analyses to detect unadjusted main effects of CpG on phenotype and using this as a benchmark. However, if sensitivity tests demonstrate an association between covariates and phenotype and/or covariates and methylation, then subsequent multi-factor models can be applied to determine the modification effect of covariates on the DMP. If the DMP signal is modified, we further recommend conducting interaction analyses, but warn that unnecessary inclusion of covariates can overburden the model and lead to reduced statistical power.

#### Differentially methylated regions (DMRs)

DMRs are genomic regions made of several contiguous DMPs. They are often associated with a specific gene region, such as CpG islands in promoter regions, but can also be in intergenic regions. Compared to DMPs, DMRs may be more biologically relevant and are more likely to be associated with modified gene expression because of the strong correlation among adjacent CpGs [65]. Therefore, accurate DMR identification is critical to enable a thorough understanding of the extent of localised differential methylation in relation to the phenotype of interest. There are multiple definitions, approaches and bioinformatic tools available for DMR identification. Thus, we recommend a workflow for identifying statistically and biologically significant DMRs using any of the bioinformatic tools reviewed below.

##### DMR identification paradigm

**Fig. 6:**
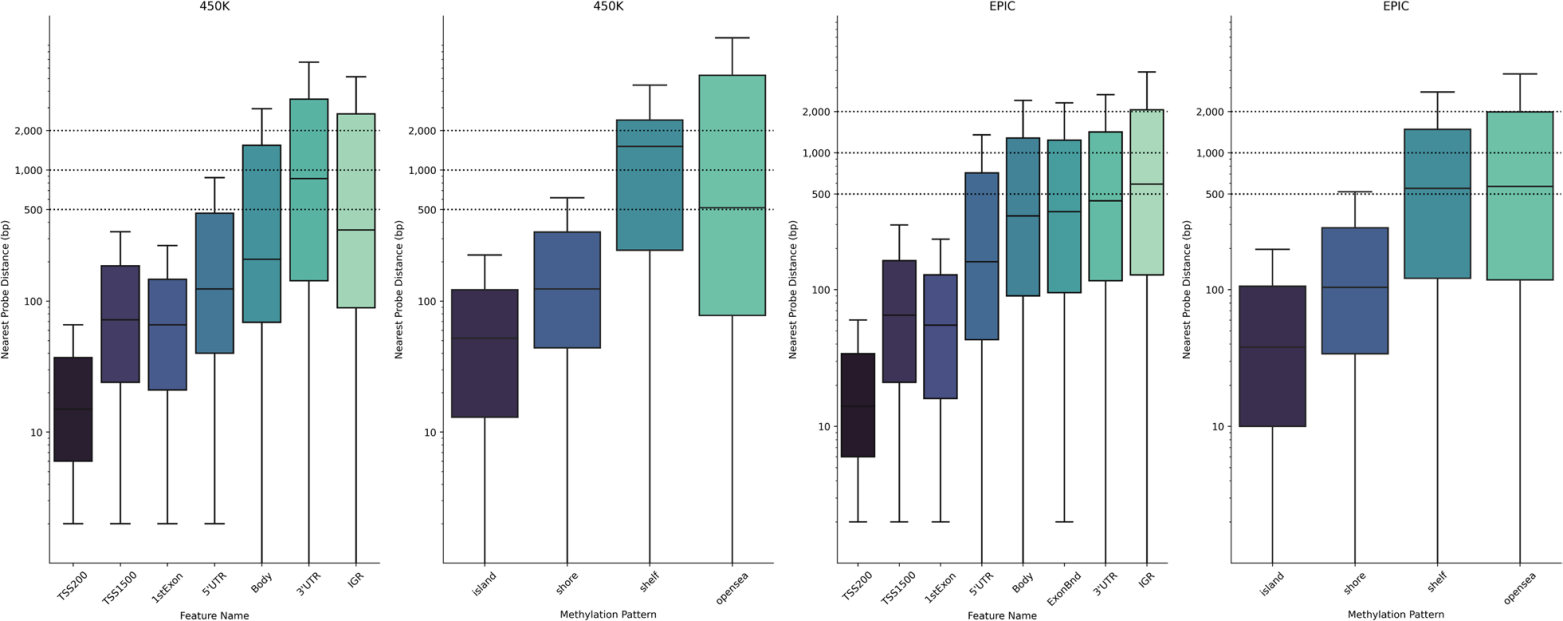
Overall distance separating adjacent probes on the 450K and EPIC microarrays. Probes were clustered based on either gene feature (TSS = transcription starting site, UTR = untranslated region, IGR = intergenic region,) or methylation pattern. Data was extracted from the probe.features.epic (EPIC) and probe.features (450K) objects provided by the ChAMP R package

A preliminary approach identifies consecutive (or closely adjacent) DMPs with the same direction of effect (i.e. all hypomethylated or hypermethylated), that yield some evidence of statistical significance based on *p* values [14, 15, 66]. For this approach we suggest filtering DMPs by an FDR < 0.05 to identify statistical evidence of association with the phenotype. This threshold is preferred over filtering based on an epigenome-wide threshold of 9.8 × 10^−8^, as it may be overly stringent and increase the risk of Type II error (false negatives). For DMR calling using this approach, researchers should decide the maximum genomic distance between DMPs (i.e. window size) and the minimum number of DMPs required for a DMR to be classified. The overwhelming majority of EWASes use a distance of 1000bp between probe to separate DMRs, regardless of the algorithm [67–70]. Some studies have applied more stringent (500bp) [71] or lenient (2000bp) windows [72]. We recommend a window size of 500–2000bp containing and at least two DMPs for a DMR to be classified, whereby the window is broadened or narrowed accordingly. For example, researchers can broaden the window size in genomic regions of low probe density. These thresholds are guided by the density of 450K and EPIC microarrays (see Fig. 6). A window of 500–1000 bp will cover most functional domains of genes, including transcription starting site (TSS) (known to modulate gene expression), 5′ untranslated region (5′UTR) and first exon. However, a window of 1000 to 2000 bp should allow researchers to identify DMRs located in region scarcely covered: gene body, 3′ untranslated region (3′UTR) and intergenic regions (IGR).

**Fig. 7:**
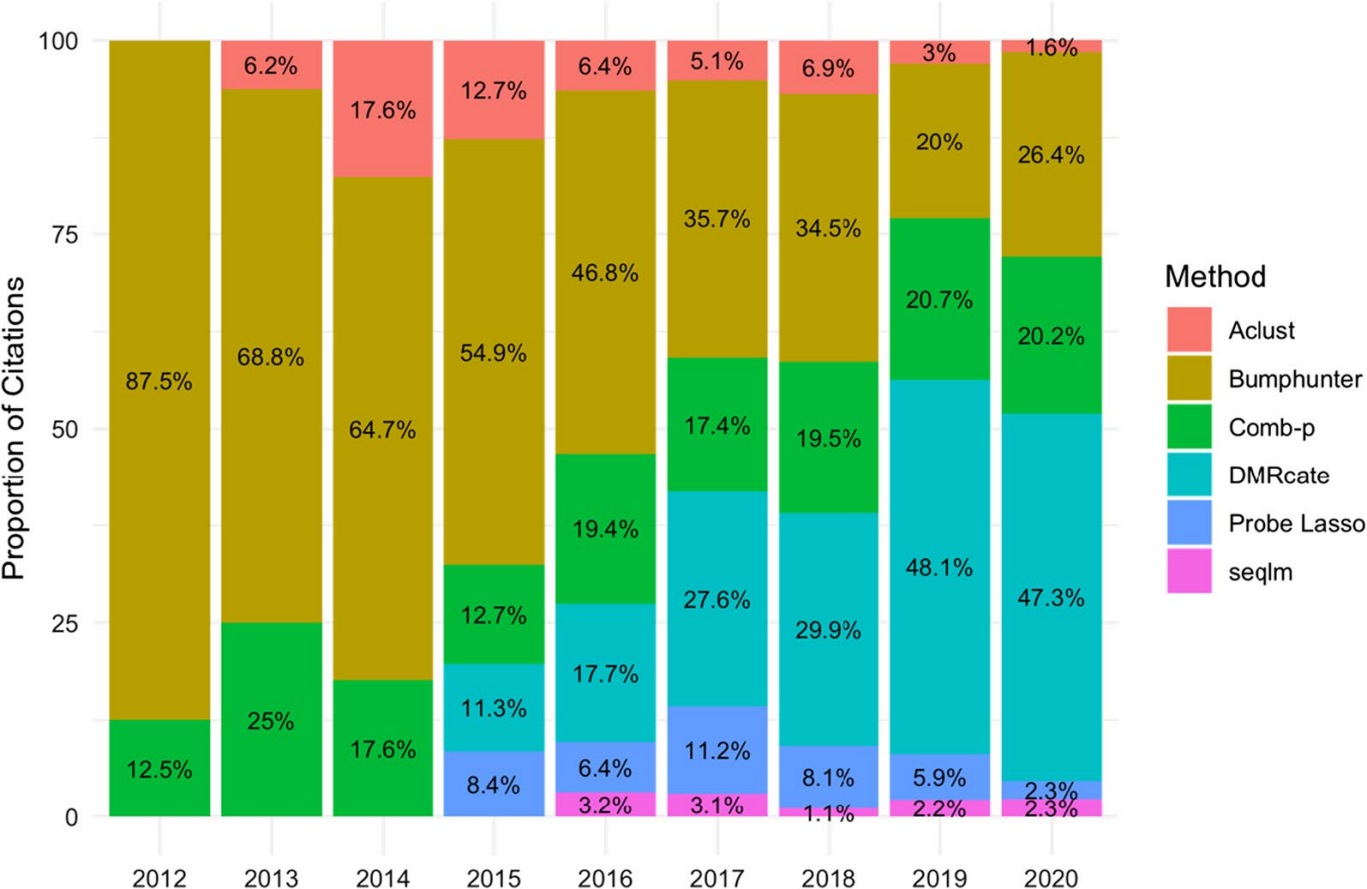
Popularity of DMR identification tools. The proportion of PubMed publications by year using common DMR identification tools. Abbreviations: DMR = differentially methylated regions

Several tools provide a more programmatic approach for identifying DMRs. Most also depend on defining the DMP threshold, genomic distance between DMPs and minimum number of DMPs per DMR. *Bumphunter* is one of the most widely used tools, created in 2012 for EWASes using high-density microarrays and large sample sizes [65]. DMRs are detected through “bump hunting” or “peak detection”. For each CpG site, *Bumphunter* produces a slope (or curve) from a linear model based on the phenotype. The smoothed curve is then plotted and analysed for “bumps” that surpass a predefined threshold. *Bumphunter*’s algorithm also incorporates covariates, and variables contributing to technical variation. However, *BumpHunter* has been shown to lack power and precision [73]. Published in 2015, *DMRcate* [74] is the most popular tool for DMR detection (as of 2021). It performs a regression of methylation level at each CpG site based on phenotype, accounting for covariates. This is followed by Gaussian smoothing (effect averaging) and grouping nearby CpG sites according to a user-defined window. Despite being more computationally intensive than *Bumphunter*, *DMRcate* outperforms *Bumphunter* and, therefore, has been the tool of choice for DMR identification since 2018 (Fig. 7). While not specific to methylation data, *Comb-p* [75] is also a popular tool to identify DMRs as its performance is comparable to *DMRcate*. *Comb-p* analyses and corrects p values in a user-defined genomic window based on weighted neighbouring probes, and then assigns a p value to the whole genomic region. Lastly*, Probe Lasso* [76] is a tool packaged with the *ChAMP* pipeline. This uses a novel variable window approach to identify larger DMRs in regions with lower probe density—such as intergenic regions. However, it does not allow covariates to be incorporated.

In a recent benchmark study comparing all primary DMR tools (*Bumphunter, Comb-p, DMRcate* and *Probe Lasso*), *Comb-p* was recommended for DMR identification as it has an adequate balance between precision and power in large and small effect sizes [73]. However, it is common for reviewers to request DMR identification with *DMRcate* if researchers use another tool. Therefore, if researchers identify DMRs with a novel tool, we recommend also using *DMRcate* for publication purposes.

##### DMR prioritisation paradigm

**Fig. 8:**
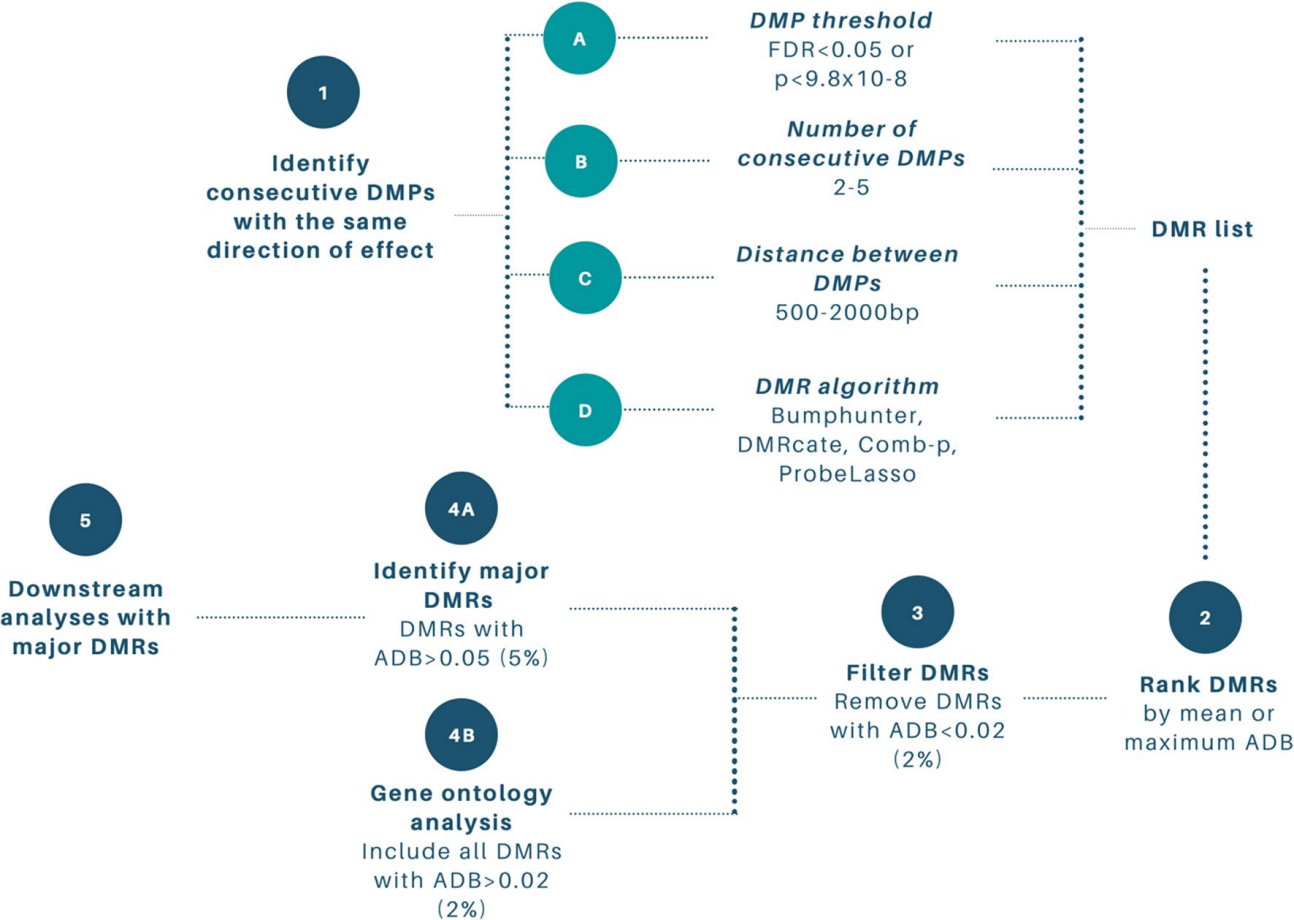
DMR identification and prioritisation paradigm. DMRs are defined as consecutive DMPs with the same direction of effect. Available bioinformatic algorithms allow researchers to select the threshold, minimum number and distance between DMPs. We recommend an FDR < 0.05, at least 2–5 consecutive DMPs, and 500–2000 bp between consecutive DMPs. After DMRs have been identified, researchers can prioritise biologically relevant DMRs by ranking them by mean or maximum absolute delta beta (ADB), filtering out DMRs with ADB < 0.02 and identifying major DMRs as those with ADB > 0.05. Major DMRs should be used for downstream functional analyses, while all DMRs with ADB > 0.02 should be used in gene ontology analysis. Abbreviations: DMR = differentially methylated regions, DMP = differentially methylated position, ADB = absolute delta beta

DMR lists contain a range of information, including mean and maximum absolute delta beta (ADB). To identify DMRs with the largest biological effects, they can be ranked by either the mean or maximum ADB. DMPs with opposite directions of effect within a single DMR can reduce the size of the mean ADB and cause important biological effects to be missed. Therefore, we recommend ranking by maximum ADB. DMRs with an ADB below 2% (0.02) should be interpreted with caution as it is difficult to discern whether these effects are caused by small, true biological effects or technical variation [6]. DMRs with an ADB > 5% (0.05) can be classified as major DMRs and used for downstream analyses, as shown in past studies [13, 14, 66]. Biological processes driving the phenotype may be a cumulative effect of several DMRs with small ADB (effect). Therefore, we recommend using all DMRs with an ADB > 0.02 (2%) for gene ontology analysis, which ideally require at least 50 genes to be informative. This paradigm is outlined in Fig. 8.

### Gene set enrichment analysis

Gene set enrichment analysis (GSEA) determines whether an a priori list of genes is enriched for specific biological terms or pathways, and the association of these with the outcome of interest. GSEA can allow biologically relevant insights to be gleaned from DMPs and DMRs. While initially developed for gene expression studies, GSEA can be applied to gene lists obtained from EWASes. Due to widespread use in genomic research, many GSEA tools have been developed and comprehensively reviewed elsewhere. Here, we will discuss just some of those that are useful for analysis of gene sets identified from EWASes.

The *ToppGene* Suite is an online tool for conducting GSEA and candidate gene prioritisation using functional annotation and/or protein–protein interaction networks [77]. *ToppGene* is a quick and easy way to perform an initial exploration of gene sets as it interrogates 14 annotation categories to detect functional enrichment of a gene list. These include gene expression, protein functional domains, protein–protein interactions, transcription factor binding sites, microRNAs, gene ontology terms (molecular functions, cellular components and biological processes), pathways, human disease phenotypes, mouse phenotypes, drug-gene association and literature [77]. Statistical associations between genes and annotation categories are tested using hypergeometric tests [77]. Notably, hypergeometric tests are unable to consider correlations between genes in the same gene set. ToppGene provides several significance thresholds for researchers to use: an unadjusted *p* value, Benjamini–Hochberg FDR, Benjamini and Yekutieli FDR and Bonferroni FDR. As per differential methylation analysis, there is no standard significance threshold for GSEA. We recommend a Benjamini–Hochberg FDR threshold of 0.05 as it balances statistical stringency with exploration of biological results, to generate hypotheses.

For GSEA with *ChAMP*, a list of genes from DMP and/or DMR analysis is required [3]. The Fisher exact test is the default method used by the function *champ.GSEA(),* but users can also select the *ebGSEA* or *GOmeth* [78] algorithm. Of these three algorithms, the Fisher exact test is the only one that fails to account for gene length bias; a phenomenon where longer genes are overrepresented in GSEA results, as they contain more CpGs than shorter genes [79]. Similarly, probe-number bias refers to the overrepresentation of genes in GSEA that contain more probes. The *ebGSEA* algorithm applies an empirical Bayes method to a normalised beta-matrix and outcome of interest [80]. It overcomes gene length bias by identifying and ranking differentially methylated genes, rather than CpGs, before performing one-tailed Wilcoxon rank-sum or known population median tests [80, 81]. The *GOmeth* algorithm overcomes gene-length and probe-number bias by correcting analyses for the number of CpGs in a gene using the *missMethyl* package [78]. It also overcomes multi-gene bias, defined as a single CpG mapping to more than one gene, leading to false positive associations. It does this with fractional weighting in the Wallenius’ non-central hypergeometric test, whereby the two genes that a CpG maps to each contribute a 0.5 weight to the intersection statistic of the test [78]. Gene-length and probe-number bias are also present in DMR analysis, whereby DMRs are more likely to be called for genes that contain more probes. There is currently only one DMR GSEA tool that addresses these biases, *GOregion*. The *GOregion* function of the *missMethyl* package [78] uses the *DMRcate* output object to identify the CpGs in each DMR, before passing them to *GOmeth*. *GOmeth* has demonstrated higher accuracy and specificity than standard GSEA tools for methylation data, including *ebGSEA* [78].

### Protein–protein interaction analysis

Protein–protein interactions (PPIs) are the basis of biological function that are affected by several factors including disease and therapeutics, and interact with molecules such as DNA [82]. PPI networks (PPINs) are mathematical representations of PPIs and can be used to understand the molecular drivers of disease states or identify potential therapeutic targets [82]. As per GSEA, a vast number of tools to identify PPINs from a gene list have been developed, some of which are discussed below. These tools utilise publicly available PPI data for which the reliability and level of annotation varies. Databases such as STRING [83] have tried to address this issue; however, it is recommended that researchers conduct PPIN analyses as an exploratory exercise, and interpret results with caution [83].

The *EpiMod* function in *ChAMP* uses the functional supervised algorithm of the *FEM* package to identify functional epigenetic modules (FEMs). FEMs are gene modules with synchronised differential methylation and expression; integrating both methylation and expression data regardless of whether the data is matched [11, 84]. Using DMP association statistics, the *FEM* algorithm identifies PPI subnetworks in the protein interactome that have a significant number of genes associated with the study’s outcome of interest [84].

The *ToppGene* application *TopGeNet* uses a list of genes to mine the protein interactome and identify genes directly or indirectly related to those on the gene list [77]. Genes that are identified are then ranked using PPIN analysis and functional annotations [77]. In PPIN analysis, genes are prioritised based on topological features of the network, quantified using PageRank with Priors, HITS with Priors and K-step Markov algorithms.

## Downstream analyses

### Methylation risk scores

The development and progression of complex diseases are polygenic. In recent years, polygenic risk scores (PRSs) have become a popular method to quantify cumulative risk of disease caused by changes with small effect sizes at multiple genes identified in GWASes. Similarly, methylation risk scores (MRSs) combine DMPs identified in EWASes into a meaningful indicator of risk at individual levels.

To construct a simple MRS, first identify DMPs associated with the trait of interest. Each DMP effect can be weighted based on their effect size. A simple MRS can be calculated for each sample as follows:

$${\text{MRS}}_{j} = \sum w_{i} \beta_{i}$$

where *w*_*i*_ represents the weight assigned to DMP, while *β*_*i*_ represents the methylation level (beta- or M values) at DMP *i* for individual *j*.

As per linkage disequilibrium in genetic studies, methylation can be highly correlated between probes (e.g. CpG islands located in promoter region of genes). Thus, removing highly correlated probes prior to MRS calculation will reduce the risk of the same signal being ascribed too much weight in the MRS. We recommend applying this filtering step prior to MRS calculation.

Simple MRSs, as well as more complex approaches, have been successfully implemented in the context of various complex diseases [85–87]. For guidance on constructing more complex MRSs, we recommend the recently published review by Hüls and Czamara [88].

### Methylation quantitative trait loci analysis

**Fig. 9:**
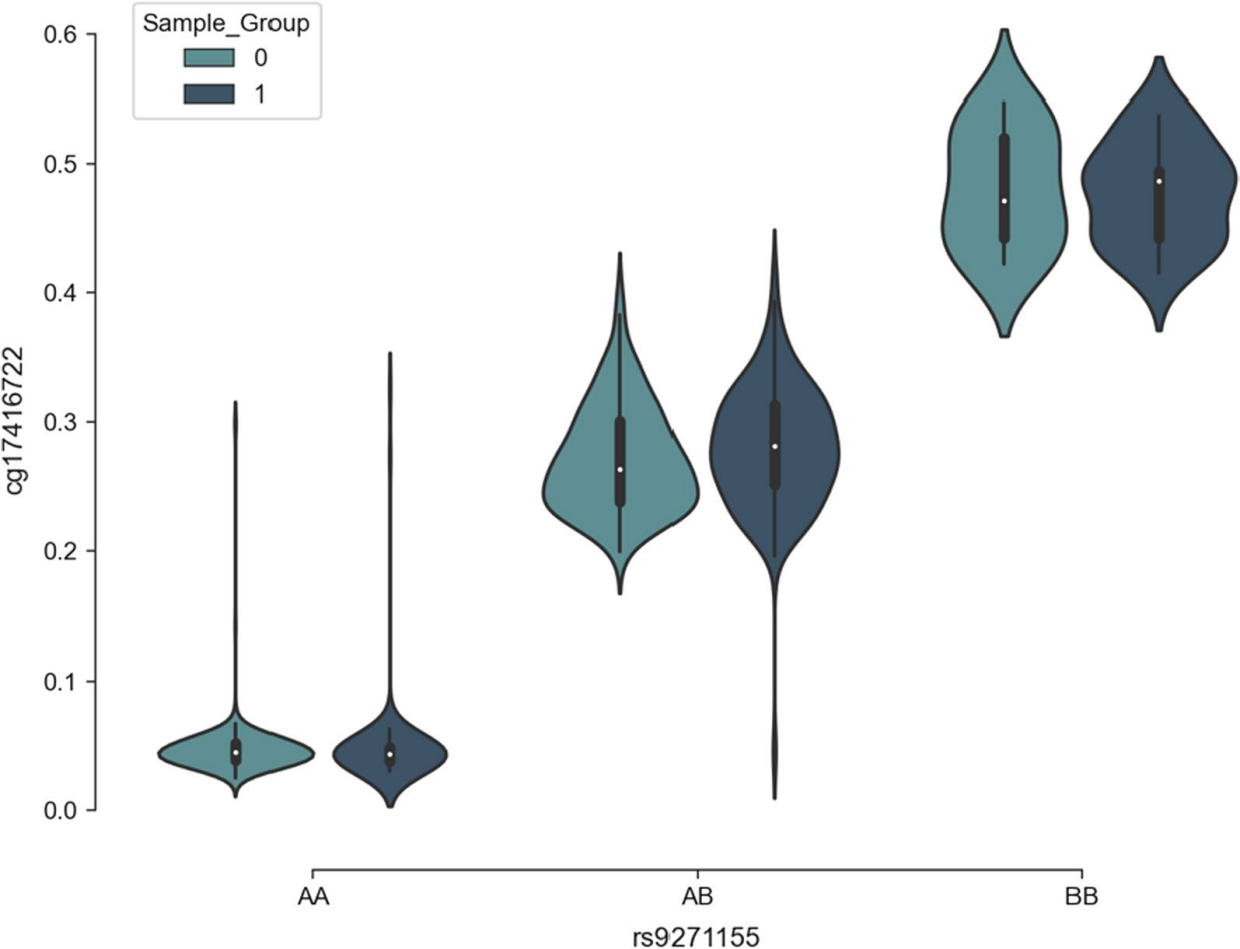
methQTL at polymorphism rs9271155 based on sample group. Genotype at rs9271155 affects methylation level at CpG site cg17416722, whereby individuals with AA genotype at rs9271155 have low methylation levels at cg17416722, and individuals with BB genotype have high methylation levels. Individuals with a heterozygous genotype (AB) have intermediate methylation levels. From [unpublished data]. Abbreviations: methQTL = methylation quantitative trait locus

Quantitative trait loci (QTL) are genomic loci influencing a quantifiable trait or phenotype [89]. The goal of QTL studies is to model the effect of genome variations on a quantifiable trait(s). QTL analysis can be used to map the relationship between methylation levels and genotype at a specific locus (methylation quantitative trait loci), and in turn determine how this relates to disease outcome (Fig. 9). Methylation quantitative trait loci (commonly abbreviated to methQTLs, metQTL or mQTLs) have been consistently identified across various diseases, populations and developmental stages [90]. There are several ways to identify methQTLs.

In a case–control cohort, one approach to identify mQTLs is to select DMPs associated with the phenotype and then select SNPs with minor allele frequency (MAF) ≥ 0.05 in the same genomic region [91]. Alternatively, researchers can select SNPs associated with the phenotype, and then select CpG sites in the same genomic region [92]. Either way, we recommend limiting the maximum window between CpG sites and SNPs, so only proximal or cis-methQTLs are identified. cis-methQTLs refer to SNPs that are associated with CpGs within the same gene. The median window size for a cis-methQTL is approximately 18kb [93]; however, common thresholds range from 50 [92] to 100 kb [94]. After feature selection, statistical modelling is commonly used to identify how the methQTLs are associated with phenotype [90].

*Plink* [95] software is commonly used to conduct GWASes. The --*assoc* command also allows researchers to relate genomic data to quantitative traits, which can include methylation levels. Using this command, users can obtain statistical model results. Notably, *plink* will compare the linear regressions of sample groups and return summary statistics if researchers encode case–control labels as covariates.

There are packages available for more advanced methQTL calculations. For example, the R package *GEM* [96] can model the complex relationship between methylation levels, genotype, covariates and one environmental variable, which could be replaced for a phenotype variable. Further, it reduces the computational time of statistical modelling by using the matrix eQTL implementation, which involves unique data pre-processing and use of large matrix operations [97].

Another method for identifying methQTLs (and QTLs in general [98]) is RUV, which uses negative controls to identify and remove unwanted variation [60, 61]. It models the association between the log of CpG methylation (Y) and factors of interests (X) while considering unwanted variation (W).

The causal inference test (CIT) [99] has also been used to identify methQTLs. CIT evaluates four different conditions that help to identify the relationship between genotype, methylation levels and phenotype. Then, for each CpG-variant pair, the relationship is classified as either: (1) null, (2) independent, (3) independent/hidden variable, (4) causal, (5) causal/independent or (6) causal/hidden. CIT is mostly targeted at continuous phenotypes but can be modified to fit categorical phenotypes, including longitudinal study time points [100].

methQTLs examine the relationship between genotype and methylation. Since this relationship is disease-agnostic and driven by genotype, it is possible to build large databases of methQTLs for researchers to leverage. The Genetics of DNA Methylation Consortium (mqtldb.godmc.org.uk), published in 2021, indexes over 270,000 methQTLs identified across 30,000 samples from over 30 cohorts [101]. As seen with eQTL databases [102], methQTL databases allow researchers to investigate the relationship between genotype and methylation in DMRs, in relation to a trait of interest.

### Estimating cell type proportions

For EWASes performed using DNA from mixed cell samples, such as peripheral blood mononuclear cells (PBMCs), it is important to consider that methylation state can vary substantially by cell subtype. Failing to address this technical characteristic with deconvolution methods can lead to two main issues, namely spurious associations between CpGs and phenotypes due to variation in cell proportion, and/or missing potentially important cell-type specific DMPs. Several statistical methods have been developed to estimate the relative proportions of the major blood cell subtypes using methylation data derived from whole blood or PBMC samples. These algorithms can be classified either as “reference-based” [103], or “reference-free” [104, 105], depending on whether they use an a priori database of cell-type specific DNA methylation reference profiles to perform deconvolution. Reference-free methods are attractive because they can, in principle, be applied to any tissue; however, the reliability of the estimates has not been well established for many cell types. Furthermore, reference-free methods do not necessarily provide individual-level estimates of cell proportions, which offer flexibility for downstream association modelling. Therefore, reference-based methods are currently the most widely used in EWASes.

The first reference-based method developed was the so called “Houseman algorithm” [103], which remains the most cited algorithm to date. Of the 182 studies published in 2020 (available on PubMed) using cell type proportion algorithms, 92.3% used the Houseman algorithm. This algorithm estimates cell proportions using a variant of regression-based calibration known as linear constrained projection (LCP), whereby non-negativity and normalization constraints on cellular proportions are imposed based on observed cell-specific data.

More recently, Teschendorff et al. [106] developed a reference-based method that used a technique called robust partial correlations (RPC). Using empirical and simulated methylation datasets, they showed that the RPC method accurately estimated proportions of the major blood cell types, and outperformed LCP in terms of error of the estimates. Thus, for accurate estimation of blood cell subtype proportions, we recommend the tool *EpiDISH*, which is available as a Bioconductor package in the R statistical environment [107]. Individual-level proportion estimates derived from *EpiDISH* can then be included in association models as covariates, and in doing so, can help identify DMPs associated with phenotypes that are independent of (or adjusted for) cell subtype variation.

### Identifying cell-specific differential methylation from mixed cell data

The methods available for identifying cell-specific differential methylation (CDM) using mixed cell data are less developed. This is mainly due to the statistical complexity of the problem, which involves interactive effects and lack of empirical data to validate statistical models. Fundamentally, identifying CDM relies on accurate cell type proportion estimation. These estimates can then be regressed against phenotypes to detect interactive effects between cell proportions and site-specific methylation signal, which indicates a CDM effect on the phenotype. Using this premise, Zheng et al. [108, 109] developed a tool called *CellDMC*. *CellDMC* takes the cell proportion estimates derived from *EpiDISH* [107] as input, and tests for interactive effects to indicate site-specific CDM signal. This method was validated using both empirical and simulated datasets, achieving >90% sensitivity and specificity. The main issue with applying *CellDMC* is the power limitation. Zheng et al. showed that sample sizes of 100 cases and 100 controls will achieve approximately 80% power to detect CDM of 20% or more [109]. However, effect sizes are typically much less than this (sometimes <5%), and therefore, CDM signals may not be detected. Fortunately, the continued reduction in costs of methylation microarrays and the availability of large cohorts from the GWAS era mean that the detection of important CDM signals is becoming more feasible.

### Methylation age acceleration analysis

Epigenetic age, or more specifically, methylation age, is a form of biological age calculated from methylation levels at CpG sites that are associated with chronological age. These CpGs are known as clock CpGs. Several algorithms, known as indices or clocks, that calculate methylation age using epigenome-wide methylation data have been created. They can be broadly categorised into chronological and biological indices. Chronological indices predict chronological age from methylation levels, while biological indices are a form of biological age that correlate with health, lifespan and clinical outcomes. Methylation age acceleration (MAA) is calculated as the residual term of regressing chronological age on methylation age, allowing researchers to compare MAA between groups or timepoints of interest.

#### Chronological age indices

**Fig. 10:**
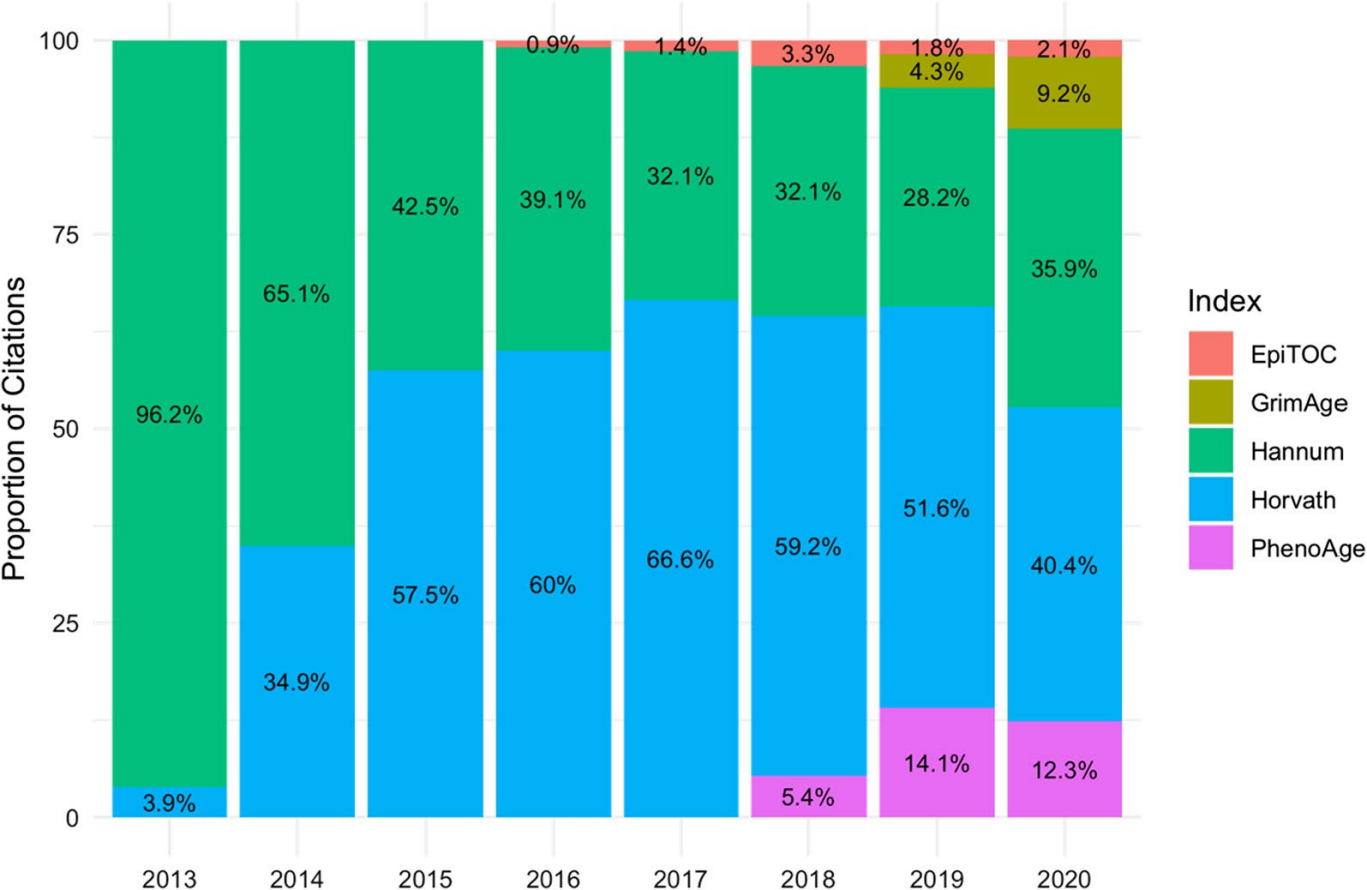
Popularity of methylation age indices. The proportion of PubMed publications by year for DNA methylation age indices

The Horvath index (2013) [110] was the first index created and is currently the most widely cited (Fig. 10). It uses methylation levels at 353 clock CpGs to predict chronological age within a five-year margin and a correlation of over 0.9 in different tissue types [110]. The strength of this index is the accurate prediction of chronological age in a range of tissues types [110]. However, tissue-specific methylation age acceleration, rather than systemic acceleration, likely occurs in many diseases. Thus, the pan-tissue nature of the Horvath index may limit its use in studies of disease-states. The Hannum index [111] was created to predict chronological age, accounting for four confounding factors gender, body mass index (BMI) and genotype [111]. It uses 71 clock CpGs to predict chronological age within 4.9 years and a correlation of 0.91 in whole blood [111]. These clock CpGs were mapped to genes associated with ageing-related conditions, metabolism and obesity [111]; suggesting a biological relevance to these clock CpGs not seen with the Horvath index. MAA using the Horvath index is associated with all-cause morbidity and mortality. However, recent studies have shown that MAA calculated with the Horvath and Hannum indices are confounded by age-related changes in cell-type proportions [112]. The main limitation of these indices that they predict chronological age exceptionally accurately and consequently, cannot identify individuals of the same chronological age but different biological ages. As biological age is more closely associated with health or disease than chronological age, it can be argued that the Horvath and Hannum indices are unlikely to provide insights into an individual's risk of, and the mechanisms driving, disease and mortality.

#### Biological age indices

The PhenoAge index (2018) [113] was developed to more accurately predict lifespan and “healthspan” by including 513 CpGs that are associated with clinical differences between individuals of the same chronological age, as well as chronological age itself. The chosen CpGs are enriched in CpG islands and Polycomb group protein targets [113]. Methylation at Polycomb group protein targets promoter regions has previously been correlated with the number of stem cell divisions, related to the stem cell theory of ageing [114]. Studies have shown that a one-year increase in PhenoAge represents a 4.5% increased risk of all-cause mortality [113], and more generally, a higher PhenoAge predicts a shorter lifespan and more age-related comorbidities (i.e. shorter healthspan) [113]. A notable limitation of the PhenoAge index is its restriction to European cohorts as there is evidence of the index tagging ethnicity, as DNA methylation is a highly heritable trait, rather than health-related outcomes.

The GrimAge index (2019) [115] is the newest methylation age index, and predicts morbidity and mortality using surrogate DNA methylation biomarkers of demographic, clinical and lifestyle variables. The GrimAge index uses methylation levels at 1030 CpGs to calculate methylation age. GrimAge can be adjusted for chronological age to obtain GrimAge Acceleration (AgeAccelGrim), which accurately predicts time to death, time to cancer and time to coronary heart disease [115]. AgeAccelGrim is further associated with T-cell senescence [116], age-related changes in blood cell proportions and leukocyte telomere length [115].

epiTOC (2016) [114] is an epigenetic mitotic clock that estimates the rate of stem cell divisions using methylation levels at 385 Polycomb group target (PCGT) promoter CpGs. As PCGT promoters are unmethylated in foetal tissue [117], age-associated hypermethylation and cumulative methylation aberrations resulting from cell divisions are used to estimate the rate of stem cell divisions [117]. The universally accelerated epiTOC rate in pre-cancerous and cancerous lesions, and epithelial tissue exposed to carcinogens, is concordant with existing knowledge of the association between cancer risk and the rate of stem cell divisions [118]. Thus, epiTOC is powerful in accurately estimating cancer risk using whole-blood methylation data [114]. The recently published epiTOC2 uses a similar method to directly estimate the number of stem cell divisions (rather than the rate), which can be used to differentiate cancer risk between tissue types [119].

For researchers to harness the accuracy of chronological age indices alongside the biological insight provided by biological age indices, we recommend calculating methylation age using a range of indices. However, the GrimAge clock deserves the most attention as this has been shown to be the single best performing index for assessing differential epigenetic ageing and predicting morbidity and mortality. In longitudinal studies, associating differences in chronological and methylation age trajectories with clinical outcomes may provide insight into disease mechanisms and risk factors. Therefore, we recommend *methyAge* function in the Bioconductor package *ENmix*, to calculate methylation age using the Horvath [110], Hannum [111] and PhenoAge [113] indices. GrimAge can be calculated using an online DNA methylation age calculator [120].

**Fig. 11:**
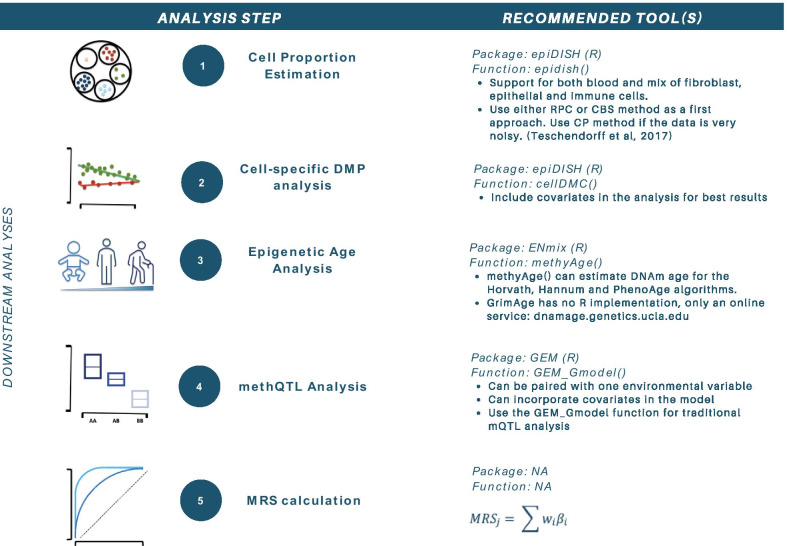
Steps and tools for downstream EWAS analyses. Recommended tools include epiDISH (https://www.bioconductor.org/packages/release/bioc/html/EpiDISH.html), ENmix (https://bioconductor.org/packages/release/bioc/html/ENmix.html) and GEM (https://bioconductor.org/packages/release/bioc/html/GEM.html). Abbreviations: EWAS = epigenome-wide association study, methQTL = methylation quantitative trait loci, MRS = methylation risk score, RPC = robust partial correlation, CBS = CIBERSORT, CP = constrained projection

Useful tools for the downstream analyses discussed above are recapitulated in Fig. 11.

## Epigenome-wide association study databases

**Fig. 12:**
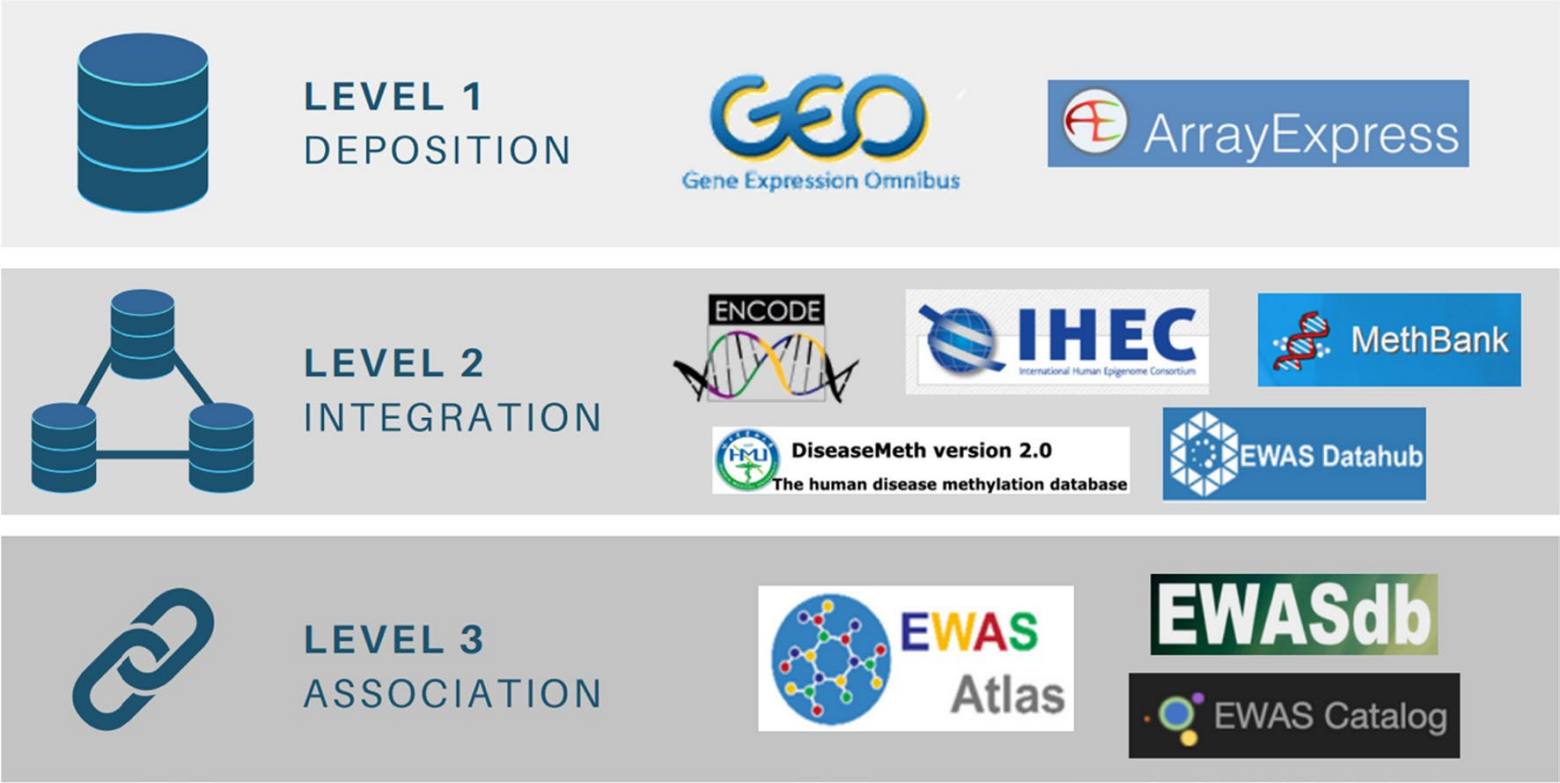
EWAS Databases containing deposited, integrated and/or associated datasets. *Deposition databases:* GEO, ArrayExpress. *Integrated databases:* ENCODE, IHEC, MethBank, DiseaseMeth, EWAS Datahub. *Association databases:* EWAS Atlas, EWASdb, EWAS Catalog. Site URLs are listed in "Packages and databases" section: Packages and databases. Abbreviations: EWAS = epigenome-wide association study, GEO = Gene Expression Omnibus, IHEC = International Human Epigenome Consortium

The number of published EWASes and associated methylation data has risen exponentially since 2009 (Fig. 1) and will continue to do so as the cost of microarrays fall. To harness this magnitude of data for scientific purposes, researchers have created databases of EWAS data. Such databases can be categorised into Deposition, Integration or Association databases (Fig. 12). *Deposition* databases contain raw methylation data and metadata, *Integration* databases contain normalised data and metadata, while *Association* databases contain normalised data, metadata and published associations at the CpG level.

*Deposition* databases, such as gene express omnibus (GEO) [121], primarily serve as data archives, as methylation data is not normalised or integrated across datasets, tissue types and diseases [121]. *Integration* databases, such as EWAS Datahub [122], have built on this by building a pipeline that performs data normalisation, batch effect correction and data standardisation across datasets [123]. *Association* databases, such as EWAS Atlas [124], further build on integration databases by including EWAS associations, manually curated from published literature [125]. EWAS databases provide a useful resource for researchers to perform analyses when laboratory resources are limited, or to validate preliminary findings. Further to this, they are valuable for untangling the molecular mechanisms underpinning methylation-trait associations, aiding in the development of diagnostic, prognostic and therapeutic tools [126].

Researchers should note that differential methylation exists among racial/ethnic groups [127, 128]. In addition, to epigenetic drift, these differences will reflect differences in both genetic and environmental factors. Of the publications reported in the EWAS Atlas, 51% represent from European cohorts, 19% African and 20% Asian cohorts [125]. Therefore, researchers should be mindful of the potential for confounding of results due to ethnic differences. Steps to guard against this include adding genomic structure components in the statistical modelling.

## Conclusion and future directions

Blood cell-based EWASes are useful experimental designs for identifying cell-type independent or cell-type specific methylation levels associated with an outcome trait of interest. The reducing cost of methylation microarrays and the advance of associated bioinformatics toolkits are aiding in the discovery of epigenetic factors related to disease outcomes. However, translating these differences into clinically useful findings, including prognostic biomarkers and therapeutic targets in the epigenome, has often been restricted by factors such as inappropriate or inadequate statistical analysis methods, insufficiently powered sample sizes, non-validated findings and an inability to establish causality. Here, we provide up-to-date recommendations for maximising the value of EWASes based on these factors. We acknowledge that our commentary is restricted to blood-based EWASes. Therefore, extrapolation of our recommendations to EWASes of other cell and tissue types should be applied with caution since there are substantive epigenetic differences. Similarly, we focus on one epigenetic mechanism, DNA methylation, without discussing histone modification and post-transcriptional regulation. These epigenetic mechanisms work together to regulate gene expression, and therefore, the study of one mechanism in isolation will limit biological understanding and the clinical relevance of results. Methods to study histone modification and post-transcriptional regulation have been reviewed previously [129, 130].

Lastly, whole-genome bisulphite sequencing (WGBS) is fast becoming a viable option for studying DNA methylation, due to recent advances in sequencing technologies. Compared to microarrays, WGBS measures methylation at a higher density of CpG sites and detects non-CpG methylation [131]. Reduced representation bisulphite sequencing (RRBS) enriches and captures DNA fragments in CpG-rich regions using restriction enzymes. The costs of RRBS and microarray-based methylation studies are comparable and affordable as they continue to fall in cost, while WGBS remains prohibitively expensive—particularly in studies that require many samples. Compared to RRBS, microarrays have more consistent genome-coverage and methylation level estimations, making them a better choice for EWASes [132]. However, RRBS is more flexible and can be used to interrogate loci that are not covered by microarrays. Third-generation sequencing technologies, such as Oxford Nanopore Sequencing (ONS), conduct long-read DNA and methylation sequencing simultaneously. Advantages of ONS include little sample preparation as bisulphite conversion is not required as with microarrays. Furthermore, ONS measures 5mC, 5hmC, 6mA methylation, while microarrays measure 5mC only.

Nevertheless, Illumina microarrays are currently 12–15% more accurate than Oxford nanopore sequencing [133], and therefore remain the most widely used sequencing technology for EWASes.

In this review, we have critically compared multiple aspects of EWAS study design and bioinformatic analysis, including numerous tools yet to be reviewed, to provide recommendations to researchers new to conducting blood cell-based EWASes.

## Packages and databases

**Table:** 

ChAMP	https://bioconductor.org/packages/release/bioc/html/ChAMP.html
Minfi	https://bioconductor.org/packages/release/bioc/html/minfi.html
Limma	https://bioconductor.org/packages/release/bioc/html/limma.html
ComBat	https://rdrr.io/bioc/sva/man/ComBat.html
Bumphunter	https://www.bioconductor.org/packages/release/bioc/html/bumphunter.html
DMRcate	https://bioconductor.org/packages/release/bioc/html/DMRcate.html
Comb-p	https://rdrr.io/bioc/ENmix/man/combp.html
PLINK	https://zzz.bwh.harvard.edu/plink/
GEM	https://bioconductor.org/packages/release/bioc/html/GEM.html
epiDISH	https://www.bioconductor.org/packages/release/bioc/html/EpiDISH.html
ENmix	https://bioconductor.org/packages/release/bioc/html/ENmix.html
missMethyl	http://bioconductor.org/packages/release/bioc/html/missMethyl.html
ToppGene	https://toppgene.cchmc.org/
FEM	http://bioconductor.riken.jp/packages/3.7/bioc/html/FEM.html
GEO	https://www.ncbi.nlm.nih.gov/geo/
ArrayExpress	https://www.ebi.ac.uk/arrayexpress/
ENCODE	https://www.encodeproject.org/
IHEC	https://ihec-epigenomes.org/
MethBank	http://bigd.big.ac.cn/methbank
DiseaseMeth	http://bio-bigdata.hrbmu.edu.cn/diseasemeth
EWAS Datahub	https://bigd.big.ac.cn/ewas/datahub
EWAS Atlas	http://bigd.big.ac.cn/ewas
EWASdb	http://www.bioapp.org/ewasdb/
EWAS Catalog	http://www.ewascatalog.org/
The Genetics of DNA Methylation Consortium	http://mqtldb.godmc.org.uk

## Data Availability

Not applicable.

## Abbreviations

ADB: Absolute delta beta
BMIQ: Beta-mixture quantile normalisation
CDM: Cell-specific differential methylation
ChAMP: Chip analysis methylation pipeline
CIT: Causal inference test
CpG: Cytosine-phosphate-guanine
DMP: Differentially methylated position or probe
DMR: Differentially methylated region
DZ: Dizygotic
EPIC: Illumina HumanMethylation850 microarray
eQTL: Expression quantitative trait loci
EWAS: Epigenome-wide association study
FDR: False-discovery rate
FEM: Functional epigenetic modules
Funnorm: Functional normalisation
GSEA: Gene set enrichment analysis
MAA: Methylation age acceleration
MAF: Minor allele frequency
methQTL: Methylation quantitative trait loci
MHC: Major histocompatibility complex
MZ: Monozygotic
PBMCs: Peripheral blood mononuclear cells
PPIN: Protein–protein interaction
QC: Quality control
RRBS: Reduced representation bisulphite sequencing
RUV: Remove unwanted variation
SNPs: Single-nucleotide polymorphisms
SSnoob: Single-sample noob
SVD: Singular value decomposition
SWAN: Subset-quantile within microarray normalisation
WGBS: Whole-genome bisulphite sequencing
27k: Illumina HumanMethylation27 microarray
450k: Illumina HumanMethylation450 microarray
